# Characterization of the bacterial microbiome of *Amblyomma scalpturatum* and *Amblyomma ovale* collected from *Tapirus terrestris* and *Amblyomma sabanerae* collected from *Chelonoidis denticulata*, Madre de Dios- Peru

**DOI:** 10.1186/s12866-022-02717-5

**Published:** 2022-12-16

**Authors:** Jesús Rojas-Jaimes, David Lindo-Seminario, Germán Correa-Núñez, Benoit Diringer

**Affiliations:** 1 Facultad de Ciencias de La Salud, Universidad Privada del Norte, Av. El Sol 461, San Juan de Lurigancho 15434, Lima, Peru; 2 DATAOMICS E.I.R.L., Lima, Peru; 3 Departamento Académico de Ciencias Básicas, Universidad Nacional Amazónica de Madre de Dios, Puerto Maldonado, Peru; 4 Incabiotec SAC, Tumbes, Peru

**Keywords:** Microbiome, *Amblyomma scalpturatum*, *Amblyomma sabanerae*, *Tapirus terrestris*, *Chelonoidis denticulata*, Peru

## Abstract

**Background:**

Ticks are arthropods that can host and transmit pathogens to wild animals, domestic animals, and even humans. The microbiome in ticks is an endosymbiotic, pathogenic and is yet to be fully understood.

**Results:**

Adult male *Amblyomma scalpturatum* (*A. scalpturatum*) and *Amblyomma ovale* (*A. ovale*) ticks were collected from *Tapirus terrestris* (*T. terrestris*) captured in the rural area of San Lorenzo Village, and males *Amblyomma sabanerae* were collected from *Chelonoidis denticulate* (*C. denticulate*) of the Gamita Farm in the Amazon region of Madre de Dios, Peru. The Chao1 and Shannon–Weaver analyses indicated a greater bacterial richness and diversity in male *A. sabanerae* (*Amblyomma sabanerae*; 613.65–2.03) compared to male *A. scalpturatum* and *A. ovale* (*A. scalpturatum* and *A. ovale*; 102.17–0.40). Taxonomic analyses identified 478 operational taxonomic units representing 220 bacterial genera in *A. sabanerae* and 86 operational taxonomic units representing 28 bacterial genera in *A. scalpturatum* and *A. ovale*. Of the most prevalent genera was *Francisella* (73.2%) in *A. sabanerae*, and *Acinetobacter* (96.8%) in *A. scalpturatum* and *A. ovale* to be considered as the core microbiome of *A. sabanerae* and *A. scalpturatum*/*A. ovale* respectively.

**Conclusions:**

We found a high bacterial diversity in male of *A. sabanerae* collected from *C. denticulata* showed prevalence of *Francisella* and prevalence of *Acinetobacter* in male *A. scalpturatum* and *A. ovale* collected from *T. terrestris*. The greatest bacterial diversity and richness was found in males *A. sabanerae*. This is the first bacterial metagenomic study performed in *A. scalpturatum/A. ovale* and *A. sabanerae* collected from *T. terrestris* and *C. denticulata* in the Peruvian jungle.

**Supplementary Information:**

The online version contains supplementary material available at 10.1186/s12866-022-02717-5.

## Background

Ticks are vectors of pathogens for different organisms and are one of the main vectors related to metaxene diseases [1]. Ticks transmit pathogenic protozoa and bacteria such as *Babesia* and *Rickettsia* respectively, can be identified by microbiological methods such as microscopy and culture in addition to molecular methods such as new generation sequencing techniques (NGS) [2, 3]. Metagenomics in ticks can identify commensal and symbiotic bacteria [4, 5] as well as pathogenic bacteria of veterinary and human interest [6]. The microbiome in ticks has yet to be explored in search of elucidating whether it has a neutral, harmful, or beneficial role for arthropods as well as for their potential hosts [6]. In this sense, previous studies on *Ixodes pavlovskyi* have described *Rickettsia*, *Anaplasma*, *Erlichia* and *Borrelia burgdorferi* and their implication in the vector and the hosts [6, 7]. Another study in *Dermacentor occidentalis* identified *Rickettsia philipii*, and two new bunyaviruses [8]. Additionally, in ticks of the genera *Amblyomma* sp., *Ixodes* sp., and *Haemaphysalis* sp., bacteria such as *Anaplasma*, *Bartonella*, *Borrelia*, *Ehrlichia*, *Francisella* and *Rickettsia* have been identified [9]. *Amblyomma scalpturatum* and *Amblyomma ovale* (*Amblyomma scalpturatum/Amblyomma ovale*) and *Amblyomma sabanerae* show a distribution in tropical forests and could be involved in the transmission of pathogens between forest animals and humans [10].

As shown, metagenomics is a very useful tool to identify potential infectious agents in ticks and to study the ecology of these agents within the framework of Public Health regarding the prevention of diseases caused by microorganisms transmitted by ticks.

Studies on microbial agents in ticks such as in western Brazil detected the presence of *Rickettsia bellii* in *A. ovale* and *A. scalpturatum* [11]. In the case of *A. scalpturatum*, it is a native tick from South America and usually parasitizes tapirs and suidae [12]. In *A. sabanerae* it has also been detected by molecular methods *R. bellii* in El Salvador [13]. Another study in Mexico, the bacterium *Rickettsia parkeri* was identified in *A. ovale* [14]. Additionally, in a multicenter study in Brazil, *R. parkeri, R. bellii, R. asemboensis* and *R. felis* were identified in *A. ovale* [15]. In Peru there is not much information on the study of ticks and their microorganisms, so our study is considered a pioneer in this type.

The objective of this study was to identify the bacterial microbiome through metagenomics in *Amblyomma scalpturatum* and *Amblyomma ovale* collected from *Tapirus terrestris* (*T. terrestris*) and *Amblyomma sabanerae* collected from *Chelonoidis denticulata* (*C. denticulata*), Madre de Dios- Peru.

## Material and methods

### Ethical aspects

This study was approved by the Office of Public Health and Environment of the Regional Council of Madre de Dios (Oficina de Salud Pública y Medio Ambiente del Consejo Regional de Madre de Dios), Peru. Laboratory procedures for bacterial identification were conducted in accordance with the international guidelines for the use of animals in research and the standards of the Animal Care and Use Committee of the Health Research Area of the Madre de Dios Regional Council Board (Comité de Cuidado y Uso de Animales del Área de Investigación en Salud de la Junta del Consejo Regional de Madre de Dios). The study was carried out in compliance with the ARRIVE guidelines.

### Geographic location

**Fig. 1:**
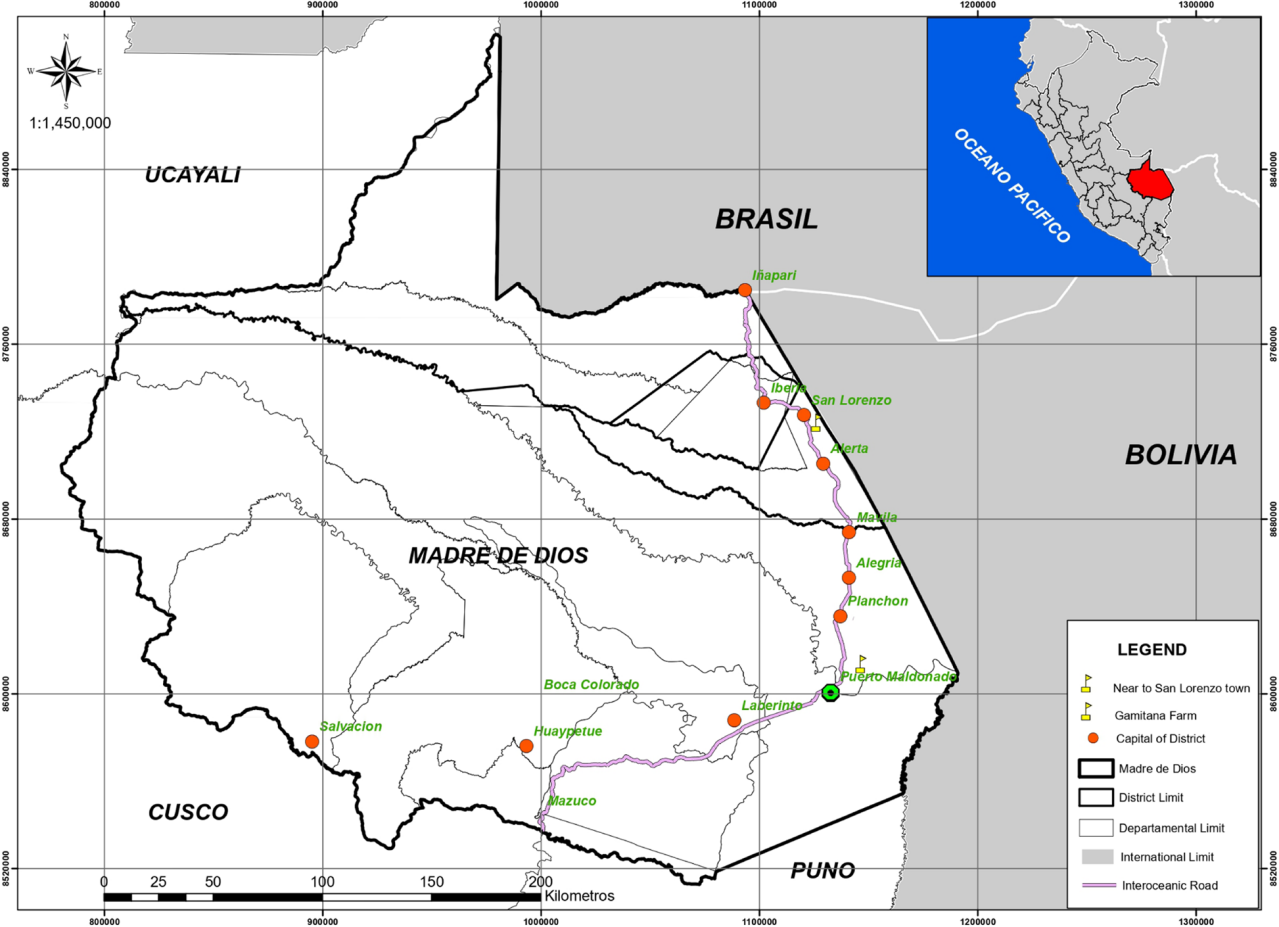
San Lorenzo Village and Gamitana Farm where *T. terrestris* and *C. denticulata* were collected respectively. This map was created with the Geoservidor https://geoservidor.minam.gob.pe/ edited with ArcGis 10.3.1 version 2015

The study was conducted in the outskirts of San Lorenzo, district of Tahuamanu ( 11° 27′ 13.73" S, 69° 20′ 2.54" W; World Geodetic System (WGS) 285 m. a. s. l), Tahuamanu province and Chacra Gamitana, district of Las Piedras (12° 30′ 36.76" S, 68° 58´ 49.3" W; WGS, 250 m. a. s. l), Tambopata province in Madre de Dios region, Peru (Fig. 1). The collection site corresponds to a forest area where hunting of wild animals is allowed. The average annual rainfall in the study area is 1,600 mm^3^, and the average annual temperature is 25 °C. The area is in the tropical wet forest zone. During sample collection, the weather was hot and humid.

### Sample collection

A wild *T. terrestris* was captured in San Lorenzo Village (11° 27′ 13,73" S, 69° 20′ 2,54" W; WGS, 285 m. a. s. l) in June 2012. 5 ticks were collected from its abdominal region 3 h after its sacrifice using forceps and were individually placed in 2 ml cryovials containing 96% ethyl alcohol. Cryovials were labeled with an identification code for the sampling site and the animal from which the sample was collected. Similarly, in the case of the Gamitana Farm, Las Piedras district, located on the left bank of the Bajo Madre de Dios River (12° 30′ 36.76" S, 68° 58´ 49.3" W; WGS, 250 m. a. s. l), manual collections of 10 “males” ticks, were taken from one *C. denticulata*. These collections were made during the daytime between 9:00 a. m. and 11 a. m.

On sterile plates ticks were washed for 15 min in a solution 0.9% isotonic sterile sodium chloride saline followed by 15 min in a solution of 96% ethanol to remove surface contaminants. Excess solution was absorbed and ticks were air-dried prior to manipulation under sterile conditions. Each tick was individually cut in half lengthwise using sterile scalpels number 15.

### Taxonomic classification

The taxonomy of the ticks was through morphological identifications using the keys of Barros-Battesti [10] at the Entomology Laboratory of the National Institute of Health of Peru in Lima (Laboratorio de Entomología del Instituto Nacional de Salud del Perú en Lima).

### DNA extraction

Total intestinal viscera DNA extraction from ticks was performed using Gentra Puregene Tissue kits (QIAGEN, Halden-Germany) according to the manufacturer’s instructions [16] from pools for each group of ticks from each animal collected (*T. terrestris* and *C. denticulata).*

### Metagenomics

To study the bacterial diversity and richness in the microbiota from ticks, the presence and quality of the extracted DNA was verified by PCR amplification of the 16S rRNA gene using the universal primers 27F (5'- AGAGTTTAGTCMTGGCTCAG-3 ') and 1492R (5'-GGYTACCTTGTTACGACTT-3') that generate a product of about 1500 base pairs (bp) [17]. All reactions were performed in 25 μl (total volume) mixtures containing 2.5 μl 10X buffer, 2.5 μl 25 mM MgCl_2_, 0.6 μl 10 mM dNTPs, and 2 U of Taq DNA polymerase (THERMO SCIENTIFIC). The PCR conditions were as follows: initial denaturation at 95 °C for 5 min followed by 35 cycles of denaturation at 95 °C for 30 s, hybridization at 55 °C for 45 s, elongation at 72 °C for 1 min, and a final elongation at 72 °C for 10 min. The PCR products were visualized by electrophoresis on a 1.5% agarose gel.

Total DNA extractions were analyzed by spectrophotometry (NANODROP EPPENDORF), and the samples with sufficient quality and quantity were shipped to MR DNA (Shallowater, TX, USA) and sequenced on the PGM platform (Ion Personal Genome Machine System, THERMO FISHER SCIENTIFIC). Metagenomic analysis was performed on the PCR amplification products of the V4 hypervariable region of the 16S rRNA gene using the 515F/806R primers [18].

#### Analysis and processing of metagenomic data

The sequences generated by Ion Torrent were analyzed with QIIME v1.9.1 [19], where the initial sequences were processed based on filtering of barcodes ≤ 6 bp, Q25 quality scores, 150 bp sequence length, and chimera detection using usearch61 [19, 20]. High-quality sequences were assigned to operational taxonomic units (OTUs) with a 97% identity cutoff for bacteria. The final OTUs were classified taxonomically using the High-Quality Ribosomal RNA Databases “SILVA” v132 database (https://www.arb-silva.de/). Likewise, unrepresentative OTUs ≤ 0.005% were filtered during analysis [21].

Lastly, the final OTUs were processed to analyze the Shannon–Weaver (SW) alpha diversity index, Chao1 richness index, beta diversity (venn and heatmap), and taxonomic abundance (barplot) of the microbial communities using the phyloseq and ampvis packages with the statistical program RStudio version 3.2.3. [19, 22, 23]. Sequences shorter than 250 bp were removed. The obtained OTUs were then taxonomically classified using BLASTn and compared with a curated database derived from Greengenes, RPDII, and NCBI (www.ncbi.nlm.nih.gov [24], http://rdp.cme.msu.edu [18]. The sequences were registered in Metagenomics Analysis Server “MG-RAST” ID: mgp98880; available at, https://www.mg-rast.org/mgmain.html?mgpage=project&project=mgp98880

## Results

### Ticks collected from Tapirus terrestris and Chelonoidis denticulata

Morphological identification indicated that ticks from *T. terrestris* belong to *Amblyomma scalpturatum* (4 males) and *Amblyomma ovale* (1 male) and ticks from *C. denticulata* belong to *Amblyomma sabanerae* (10 males) in Madre de Dios [10]. The ticks were collected in San Lorenzo Village and Gamitana Farm respectively (Fig. 1).

### Statistical values and diversity in Amblyomma scalpturatum/Amblyomma ovale and Amblyomma sabanerae

Microbiome analysis using the 16 s-515F/16 s-806R primers and amplicon sequencing on Ion Torrent PGM (Ion Personal Genome Machine System, THERMO FISHER SCIENTIFIC) generated a total of 173,945 raw reads (86,972.5 average) from the two analyzed samples [16–18] (Table 1). After rigorous data curation, 96,696 high-quality sequences were retained with an average of 48,348 sequences per sample and an average length of 150 bp and quality > 25 [19, 20]. The maximum number of filtered sequences number of assigned sequences OTUs 66,792 was obtained in the mix from *Amblyomma scalpturatum* and *Amblyomma ovale*, which exceeded those found in *Amblyomma sabanerae* by 223.4% [21]. These sequences were assigned to 564 total unique sequences corresponding to 282 abundant (< 0.005%) OTUs based on a > 97% identity cutoff for bacterial 16S rRNA genes [21]. At the individual sample level, the microbiome from *Amblyomma sabanerae* surpassed that from the mix from *Amblyomma scalpturatum* and *Amblyomma ovale* (478 and 86 OTUs, respectively). At the taxonomic level, a total of 28 genera distributed in 25 families, 17 orders, 10 classes, and 7 phyla were detected in the mix from *Amblyomma scalpturatum*/ *Amblyomma ovale* and 220 genera distributed in 134 families, 68 orders, 35 classes, and 20 phyla were detected in *Amblyomma sabanerae* respectively*.*

**Table 1:** Statistical summary of the microbiome of *Amblyomma scalpturatum*/*Amblyomma ovale* and *Amblyomma sabanerae*

Sample characteristics	*Amblyomma scalpturatum*/*Amblyomma ovale*	*Amblyomma sabanerae*
Total, number of sequences	114.845	59.100
Number of filtered sequences	47.588	27.756
Number of assigned sequences OTUs	66.792	29.904
Number of OTUs	86	478
Phylum	7	20
Class	10	35
Order	17	68
Family	25	134
Genus	28	220
Richness and diversity índices	*Amblyomma scalpturatum*/*Amblyomma ovale*	*Amblyomma sabanerae*
Chao1	102.17*	613.65*
Shannon	0.46*	2.03*

^*^Significant differences (*P* < 0.05)

The SW index reflects the specific diversity of each sample, whose value increases as the number of different OTUs increases. In this study, the microbiome obtained from *Amblyomma sabanerae* ticks samples showed a higher SW index than the in the mix from *Amblyomma scalpturatum* /*Amblyomma ovale* microbiomes. On the other hand, Chao1, the index that evaluates specific richness, showed that the number of expected OTUs decreased from 613.65 in *Amblyomma sabanerae* to 102.17 in the mix from *Amblyomma scalpturatum*/*Amblyomma ovale* after the standardization of the sample size to 12,364 sequences. Statistical analyses of variance of the SW and Chao1 indexes in the *Amblyomma sabanerae* and in the mix from *Amblyomma scalpturatum* and *Amblyomma ovale* samples showed significant differences (*P* < 0.05) [22–24].

### Composition of the core and shared and individual microbiome from *Amblyomma scalpturatum*/*Amblyomma ovale* and *Amblyomma sabanerae*

The comparative analysis of the composition of the microbiota from *Amblyomma scalpturatum*/*Amblyomma ovale* and *Amblyomma sabanerae* revealed that 8.8% out of the 228-genus found in mix from *Amblyomma scalpturatum*/*Amblyomma ovale* and *Amblyomma sabanerae* were common in the two groups. This shared community is considered as the core microbiome of *Amblyomma* ticks. The percentages showed a decreasing proportionality in *Amblyomma sabanerae* and *Amblyomma scalpturatum*/*Amblyomma ovale* in relation to the non-shared bacterial genus.

### Microbiota between twenty-three most prevalent bacterial genera in *Amblyomma scalpturatum*/*Amblyomma ovale* and *Amblyomma sabanerae*

**Fig. 2:**
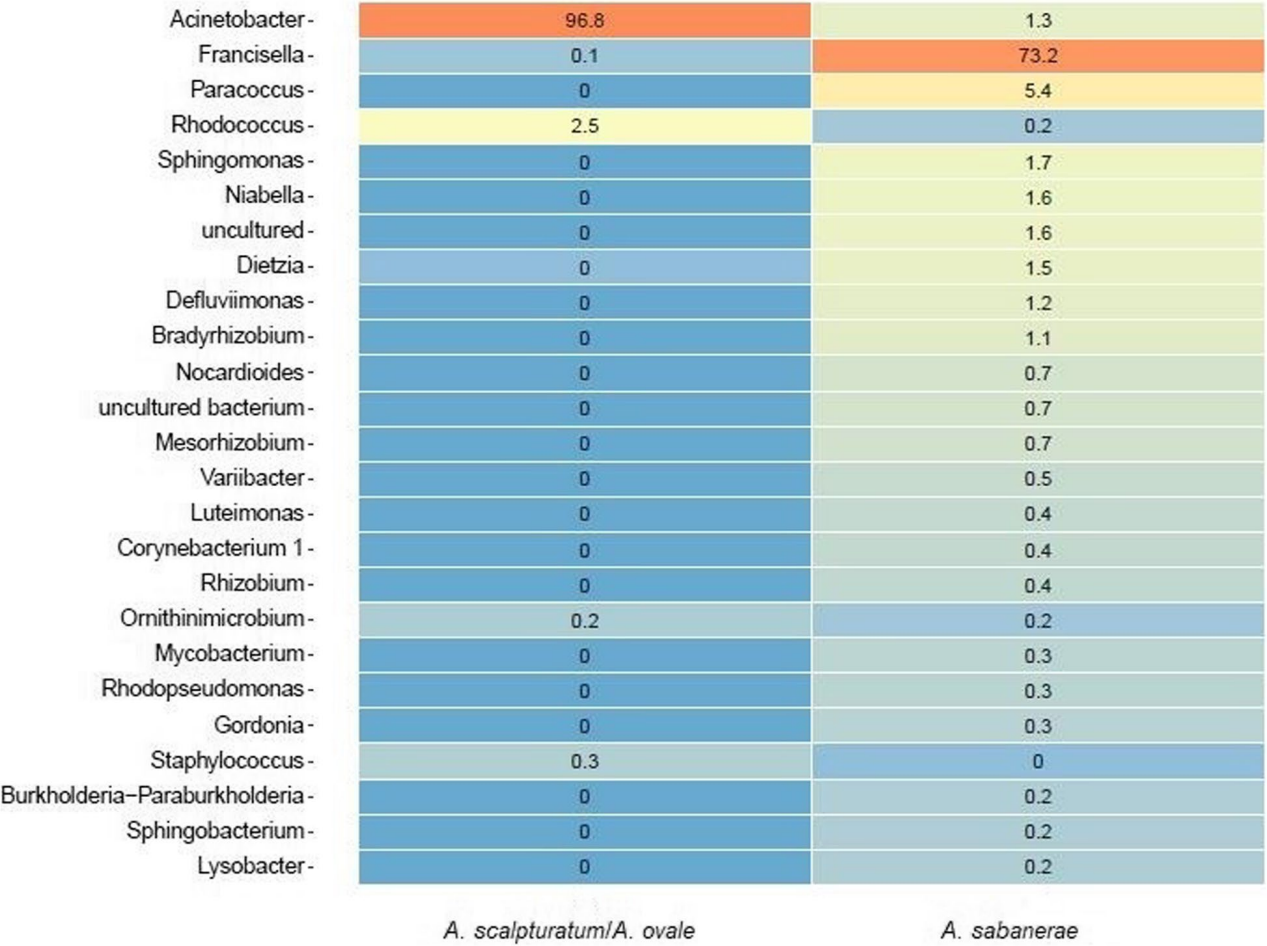
Microbiome abundance according to *Amblyomma scalpturatum*/*Amblyomma ovale* and *Amblyomma sabanerae.* (Rstudio version 3.2.3. https://cran.rstudio.com/bin/windows/base/old/3.2.3/)

Regarding the abundance of bacterial genera in *Amblyomma scalpturatum*/*Amblyomma ovale*, *Acinetobacter* was the most abundant genus (96.8%), while *Francisella* was the most abundant genus in *Amblyomma sabanerae* (73.2%) (Fig. 2).

### Microbiome richness estimation in Amblyomma scalpturatum/Amblyomma ovale and Amblyomma sabanerae

**Fig. 3:**
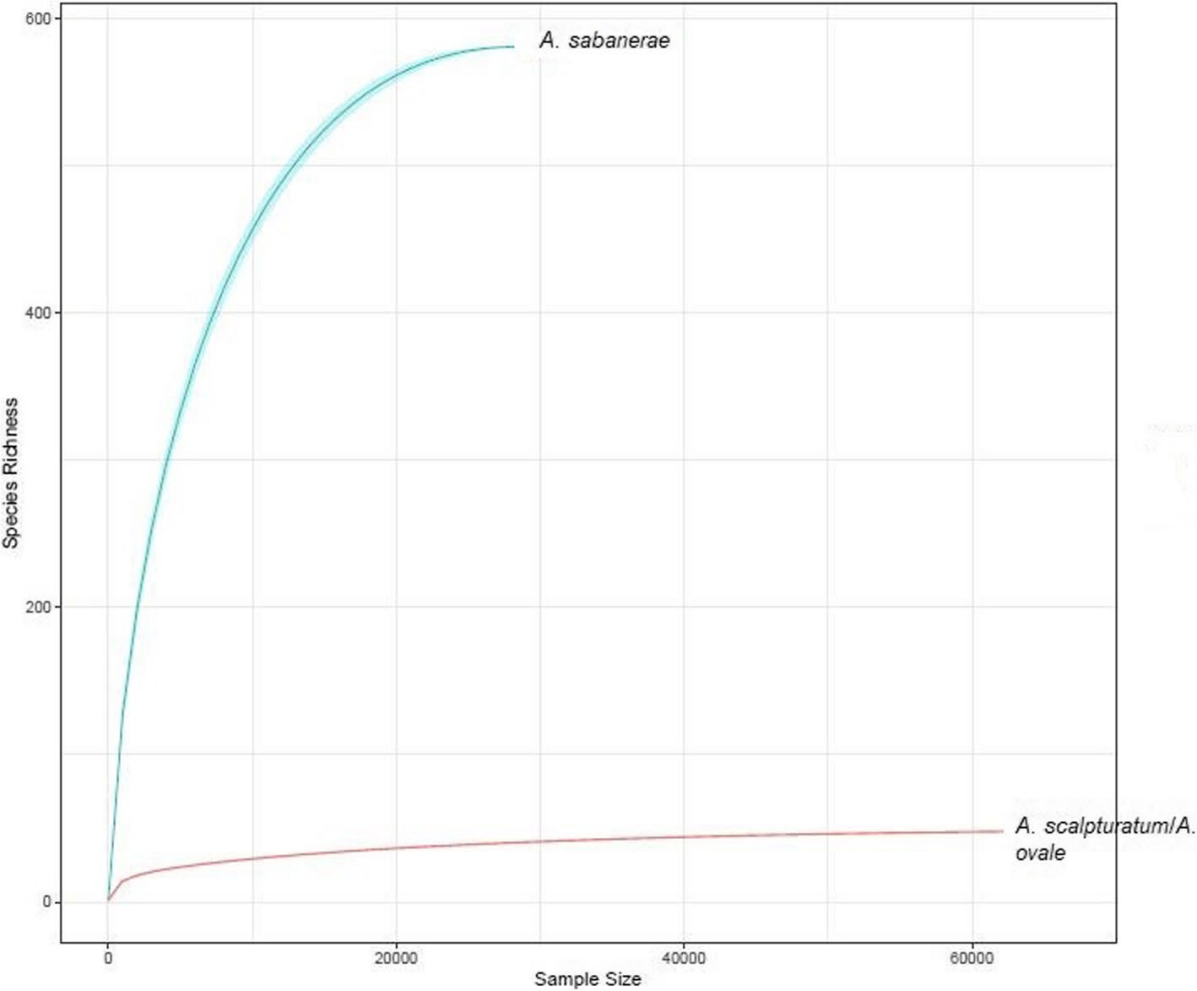
Rarefaction curves representing microbiome richness presents in *Amblyomma scalpturatum*/*Amblyomma ovale* and *Amblyomma sabanerae*, collected from *T. terrestris* and *C. denticulata.* (Rstudio version 3.2.3. https://cran.rstudio.com/bin/windows/base/old/3.2.3/)

The analysis of the rarefaction curves illustrates the differences obtained between the high OTUs number with a high richness and biodiversity of 613.65 and 2.03 respectively at a lower sequencing depth (29,904 assigned reads) of *Amblyomma sabanerae* compared with a lower richness at a higher sequencing depth (66,792 assigned reads) of *Amblyomma scalpturatum*/*Amblyomma ovale* (Fig. 3). Nevertheless, rarefactions curves also show a plateau phase profile that indicates that most of the OTUs present in the samples have been identified.

## Discussion

The richness and diversity indexes revealed that the microbiota present in *Amblyomma sabanerae* exhibit greater bacterial genera diversity and richness than the microbiota in *Amblyomma scalpturatum*/*Amblyomma ovale*. Previous studies in ticks of *Ixodes ovatus, I. persulcatus,* and *Amblyomma variegatum* have shown differentiated microbiome profiles both at the taxonomic and functional levels between sexes of the same tick species [25], however in our study all ticks (4 *A*. *scalpturatum)*, (1 *Amblyomma ovale*) collected from *T. terrestris* and (10 *A. sabanerae*) collected from *C. denticulata* were males*.* Therefore, the difference in the microbiome by sex was not determined.

In addition, geographical location, temperature, humidity, species, sex, anatomical location, and type of diet have been shown to affect the microbiome of ticks [26–31]. In our study, although ticks were of the same genus, statistically (*p* < 0.05) significant differences were found in bacterial diversity and richness related to the animal collected, in the case of *A. sabanerae* collected from the *C. denticulata* reported higher richness and diversity (613.65–2.03) compared to *A. scalpturatum*/*A. ovale* (102.17–0.46) collected from the *T. terrestris* respectively.

Among the 228 different genera identified, the core microbiome that included the majority of the most prevalent genera stood out. Several of the identified genera within the core microbiome are known to be human pathogens (*Streptococcus, Francisella, Pseudomonas, Staphylococcus, Acinetobacter*). *Staphylococcu*s, is mainly related to infections in soft tissues and has been previously reported in the gut of *R. microplus* and with a high prevalence in female *Amblyomma variegatum* [9, 25], *Pseudomonas* has been suggested to be involved in the infection of soft tissues, including the tissues of the respiratory system [32, 33]. *Streptococcus* is bacteria can cause many diseases ranging from mild skin infections to respiratory infections [34].

In addition, a moderate bacterial microbiome was shared between *A. sabanerae* and *A. scalpturatum/A. ovale* ticks [20 (8.8%)] compared to the specific bacteria genus in *A.scalpturatum/A. ovale* [8 (3.5%)] and *A. sabanerae* ticks [200 (87.3%)]. We suggest that these differences have a behavioral origin related to the host (*T. terrestris* and *C. denticulata*) [11–13]. Thus, female and nymph ticks are more prone to remain on the same host, whose microbiota impact on the tick gut microbiome, while male ticks frequently change hosts as our case where all ticks were male [25]. This hypothesis is supported by studies on other genera that reported higher relative abundance and alpha diversity in female ticks than in male ticks, however in our case we cannot compared genders because all ticks were males so the richness and diversity will be because the males *A. sabanerae* collected from *C. denticulata* change host that are linked to different genus of reptiles compared male *A. scalpturatum* collected from *T. terrestris* which are more specific for its host [13–15, 35, 36]. Additionally, it is necessary to consider that the role of nuclei bacterial genera and the species included in these may present different roles as pathogens or symbionts depending on whether they are found in the arthropod or in the vertebrate that hosts the arthropod.

The most prevalent bacterial genus among of *A. scalpturatum/A. ovale* was identified as *Acinetobacter* (96.8%), whose members cause infections at the level of the respiratory, urinary system and wound, in addition this bacterium tends to acquire resistance to various antibiotics and is of importance in Public Health, especially at the hospital level, *Acinetobacter* has been reported in a metagenomic study in *I. persulcatus, I. pavlovskyi,* and *Dermacentor reticulatus* [37]. *Rhodococcus* (2.5%) the second most abundant genus in *A. scalpturatum/A. ovale* has the ability to metabolize a large number of substrates and cause pulmonary infections, especially in immunocompromised people [38].

In *A. sabanerae, Francisella* (73.2%) was the most prevalent bacterial genus. Regarding the role of bacteria in ticks, note that nonpathogenic microorganisms present in ticks could cause infections in humans and other animals. For example, ecological studies have shown that *Rickettsia, Francisella,* and *Coxiella*, which are considered vertebrate pathogens, can change their pathogenic role and have a mutualistic and symbiotic relationship with ticks [1]. In the case of *Francisella* it is considered as a representative genus of endosymbionts related to pathways for biotin, folic acid, and riboflavin biosynthesis and it is found on rare occasions in some ticks and in the case of *F. tularensis* as a causal agent of tularemia, a very contagious and life-threatening disease [1, 39, 40]. Therefore, studying the interaction between the bacterial microbiota and ticks is of utmost importance for the control of pathogens [1]. Symbiotic bacteria as *Coxiella* sp. and *Francisella* sp. are linked to the synthesis of vitamins necessary for the survival of *Amblyomma* and *Rhipicephalus* [41–45]. Likewise, other symbiotic bacteria, such as *Rickettsia*, and *Rickettsiella*, have been reported in ticks [39]. However, in our study not *Coxiella* neither *Rickettsia* was found.

*Paracoccus*, the second most abundant genus (5.4%) in *A. sabanerae*, is a coccobacillary bacterium that is typically present in a wide range of ecosystems and this bacterium is considered by its diversity of metabolic production in different ecological environments and with biotechnological interest [46].

In our case, *A. scalpturatum* that is found only in South America is a tick that mainly parasitizes *T. terrestris.* This tick exhibits host specificity so this is the reason for the specificity microbiome, sometimes *A. scalpturatum* can bite human and is related to transmit *Rickettsia* [11, 12]. *A. scalpturatum* was found in *T. terrestris*.

*T. terrestris* was likely infected by ticks in the jungle and them could infect human due to the proximity of San Lorenzo Village, where livestock farming and hunting are practiced. Previous studies highlight that *A. scalpturatum* can infect *T. terrestris*, *Pecari tajacu*, and humans [11, 12], with the potential risks of pathogen transmission that this implies. In the case of *A. ovale* collected from *T. terrestris* can parasite many mammals as *P. tajacu* and can transmit *Rickettsia* and is found predominantly in sylvatic areas [14, 15]. In our study it was found parasitizing *T. terrestris.*

*A. sabanerae* was collected from *C. denticulata*. *A. sabanerae* can parasite different types of reptile as turtle, a previous study showed *Rickettsia* in *A. sabanerae* collected from a turtle (*Kinosternon* sp). In our study *A. sabanerae* was found in a turtle (*C. denticulata*) in the Chacra Gamitana Village where the farming is practicing, so the farmer could be parasite by the ticks and infected with some pathogenic bacteria as *Rickettsia* and *Francisella* [13, 35, 36].

According to previous studies, the endosymbiont bacteria of a species of tick vary depending on the ecology and the number of ticks studied [46]. Therefore, the importance of our study is the finding of the new microbiome of *A. scalpturatum/A. ovale* collected from *T. terrestris and A. sabanerae* collected from *C. denticulata.*

The small number of ticks was justified by the fact that *Amblyomma* ticks are not very common studied on the wild host *T. terrestris* and *C. denticulata* in the jungle of Madre de Dios-Perú; therefore, we could not collect a larger sample of ticks. Our interest was to study the microbiota of ticks as *A. scalpturatum/A. ovale* and *A. sabanerae* that parasitizes *T. terrestris* and *C. denticulata* respectively and who live in the Peruvian Amazon.

Among the limitations of our study is the bacterial microbiome found in 5 males of ticks collected from *T. terrestris* and 10 males’ ticks collected from *C. denticulata*, which implies a bacterial microbiome representative of a specific circumstance and ecology. Therefore, studies with a greater number of samples could show a greater diversity of species and different percentages of bacterial abundance.

## Conclusion

In this study, we found a high bacterial diversity in male of *A. sabanerae* collected from *C. denticulata* showed prevalence of *Francisella* and prevalence of *Acinetobacter* in male *A. scalpturatum/A. ovale* collected from *T. terrestris*. The greatest bacterial diversity and richness was found in males *A. sabanerae.* This is the first bacterial metagenomic study performed in *A. scalpturatum/A. ovale* and *A. sabanerae* collected from *T. terrestris and C. denticulata* in the Peruvian jungle. This study lays the foundations for future studies on the importance of the role of the identified bacteria on arthropods and animal and human health.

## Supplementary Information

**Additional file 1.**

## Data Availability

All data generated or analyzed during this study are included in this published article. Raw data are available. The preprocessing, statistics, OTUS, abundancy, taxonomy, diagram of Venn are included in the Supplementary Text. The authors confirmed that all supporting data have been provided within the article or through supplementary materials.

## Abbreviations

GART: Ticks collected from *C. denticulata*
GARS: Ticks collected from *T. terrestris*
m. a. s. l.: Meters above sea level
PCR: Polymerase chain reaction
SW: Shannon–Weaver
OTU: Operational taxonomic units
PGM: Ion Personal Genome Machine System
NGS: Next-generation sequencing
rRNA: Ribosomal RNA
WGS: World Geodetic System
SW: Shannon–Weaver
NCBI: National Center for Biotechnology Information
pb: Base pairs
